# Fantasy Sexual Material Use by People with Attractions to Children

**DOI:** 10.1007/s11920-023-01435-7

**Published:** 2023-07-31

**Authors:** Rebecca Lievesley, Craig A. Harper, Ellie Woodward, Gilian Tenbergen

**Affiliations:** 1 NTU Psychology, School of Social Sciences, Nottingham Trent University, Nottingham, UK; 2 Department of Psychology, State University of New York at Oswego, Oswego, NY 13126 USA

**Keywords:** Sexual fantasy, Pedophilia, Sexual abuse prevention, Fantasy sexual materials (FSM)

## Abstract

**Purpose of Review:**

With the Internet allowing consumers easy access to fantasy and fictional sexual materials (FSM), it is becoming increasingly important to understand the context of their use among specific populations. Of particular, social, clinical, and legal interest is FSM use by people who are attracted to children and whether this may have a risk-enhancing or protective impact on their likelihood of committing a contact or non-contact sexual offence.

**Recent Findings:**

There is a lack of data currently available in relation to the use of FSM by those with sexual attractions to children. Evidence from allied areas appears to show no meaningful associations between FSM use and sexual aggression.

**Summary:**

We propose a novel research program and some initial research questions that provide a theoretical framework for more evidence-based inquiry on FSM use by people who experience attractions to children.

## Introduction

Fantasy or fictional sexual materials (FSM) can take a variety of forms, from written stories to silicone sex dolls, all with the purpose of instigating (e.g., through engaging in fantasizing) or enhancing (e.g., through masturbatory practices) sexual arousal and fulfillment [1, 2]. Much remains unknown about access to and the utilization of such materials, especially among those living with sexual attractions to children within the community [3•, 4]. Despite the potentially revolutionary effects that developing an understanding of FSM use in the population might have in clinical or abuse prevention contexts, there is a lack of empirical data currently available.

This lack of data has not prevented a burgeoning public policy response to the issue, namely in the form of policing fictional outlet sites and their content [5, 6]. Here, we explore the potential uses of FSM among those who experience sexual attractions to children, setting out the myriad ways that FSM might play a role in the sexual and emotional health of this population. In addition to this, we discuss the potential for FSM to play a role in efforts to prevent sexual offending by those who may, by some, be considered to be at an increased risk of engaging in abuse. We close the paper by articulating a set of research questions and broad study areas to progress this important emerging area.

## Understanding Sexual Attractions to Children

There is a growing interest in understanding the nature, expression, and management of sexual attractions to children within the social scientific literature. This body of work contains several labels to describe these attractions, including “pedophilia” [7•, 8–10], “pedohebephilia” [11, 12], “minor attraction” [13–16], and “child attraction” [17, 18]. Although these terms are often used interchangeably [19], they are not always conceptually equivalent. In talking about sexual attractions to children, we invoke Michael Seto’s notion of chronophilias, which describe discrete (though often overlapping) patterns of sexual attraction to specific age groups or developmental stages [20••]. These range from nepiophilia (attractions to very young infants) through pedophilia and hebephilia (attractions to prepubescent and pubescent children, respectively), and finally ephebophilia and teleiophilia (attractions to postpubescent teenagers, and finally to adults). Chronophilias also extend past attractions to those of typical reproductive age, with mesophilia (attractions to middle-aged adults) and gerontophilia (attractions to older adults) completing the taxonomy.

Although sexual attractions to children can act as a motivating factor for abuse perpetration [21], many individuals who experience such attractions will never commit any sexual offences [7•]. Estimates suggest that roughly 25–50% of incarcerated individuals who commit child sexual abuse offenses are primarily or exclusively motivated by attractions to children, which serves as further data to cast doubt over a simplistic association between attractions to children and the committing of sexual abuse [22]. When referring to [people with] sexual attractions to children in this paper, we adopt a holistic view of the broad spectrum of such sexual arousal patterns (i.e., from nepiophilia to ephebophilia, whether with or without additional attractions to adults), and make no assumptions related to associated offending behaviors. As such, we are directly interested in exploring the potential uses of FSM among this population in a range of personal and clinical contexts.

## Fictional Sexual Materials

There is no currently accepted definition of fictional sexual materials (FSM), but broadly described these can be any type of outlet that does not include physical contact with a real person and that results in sexual arousal and/or gratification [5]. Although FSM might sometimes depict a real person (e.g., in the form of an image, story, or mentalized sexual fantasy), the content of such material is not based in reality. The lack of operationalization of FSM within the broader literature leads to difficulties in discussing this topic. For example, scripted adult pornography could be considered fictional, as models within the material are often playing roles. However, other forms of pornography, such as those typically found in “amateur” categories, may depict real couples engaging in real sexual activity. As such, these specific materials would not necessarily be considered FSM due to this degree of reality (though future research might determine that the impersonal nature of watching such material shifts it into the FSM category). A non-exhaustive set of more definitive examples of FSM might therefore include (child-like) sex dolls, virtual/augmented reality avatars and characters, self-generated fan fiction stories involving known characters/actors, and self-generated artistic drawings/stories [1, 2].

The legality of such materials varies greatly across the globe. For example, Australia criminalizes the use of written and visual materials that are thought to relate to children [5, 6]. In the USA, federal law criminalizes sexual material depicting children (whether real or fictional) under obscenity laws, though a plethora of legal challenges to this law have been brought forth in recent years. On the topic of child-like sex dolls, there is currently no federal law in place to regulate their access or use; however, individual states have begun introducing legislation to address this gap [23]. In the UK, ownership of a child-like sex doll is not currently criminalized, but importing or posting a child-like doll is illegal under postal laws. Given this lack of consistency in the law across different jurisdictions, we are less focused on the legality of specific forms of FSM in this article. Instead, we place our emphasis on the psychological functions that such sexual materials serve for people who are attracted to children.

What differentiates FSM from other forms of sexual expression is their basis in fantasy or fiction, meaning that a person can imagine themselves in a sexual or romantic situation with an individual with whom actual activity and behavior could be either illegal or harmful if acted out in the real world. Considering this, FSM may have clinical utility in their use by those with attractions to children, given that acting on one’s sexual attractions when they involve a child is illegal, and can result in lasting psychological trauma for the victim [24, 25]. There is little that has been written specifically on the topic of FSM in relation to unique populations such as those who are attracted to children. Until this is understood, it remains difficult for clinicians to structure support services or treatment interventions in line with the needs and goals of an individual (which may include dealing with sexual frustration) [14, 26••]. The following review will highlight general knowledge about FSM, with specific reference to the target population when available.

### Defining Sexual Fantasy

Sexual fantasy is a form of mental thought and/or imagery that initiates or enhances sexual arousal [27, 28]. Recent conceptualizations using the Dual Process Model for Sexual Fantasizing (DPM-SF) [29••] break down sexual fantasy into both of its constituent components: the content of sexual fantasy and the process of sexual fantasizing. Bartels and colleagues stress the importance of assessing both components, as without a deeper understanding and awareness of both content and process, an overinflation of the importance of fantasy content can occur [29••]. For example, one might consider the fact that somebody masturbates to sexual fantasies involving children to be problematic *because of the content of those fantasies*. However, if the process of fantasizing causes no harm (to others) or impairment/distress (to the person with such fantasies), then the problematic nature of the fantasy becomes less clear-cut.

There is also a distinction to be drawn between fleeting sexual thoughts and actively constructing, rehearsing, and masturbating to vivid mental scenarios [29••]. The former is hypothesized to be a relatively common experience that occurs situationally and involuntarily in the course of one’s everyday life. For instance, somebody may see a sexually attractive stranger on a mode of public transport and experience a fleeting bout of sexual arousal. These automatic processes are instinctive and are not the focus of our discussion in this paper. On the other hand, the latter form of fantasizing is suggested as a more selective experience that involves a planned and intentional action. This conceptualization is relevant for our discussion of the use of fictional sexual material (FSM) since, according to the DPM-SF, it should not be only the mere *use* of material that is considered both clinically and scientifically, but also the *frequency*, *duration*, *intensity*, and *function* of such use. It is also in this more experiential detail (e.g., whether fantasy engagement leads to disruptions in everyday functioning, personal well-being, or offending risk) that it is possible to determine whether fantasy engagement can be classified as problematic [30]. Without a more nuanced approach to understanding both what FSM are and how they are used, in this specific case by those with attractions to children, much is lost to fear of the “what if?” and hypothetical arguments related to an escalation towards sexual offending.

### Sexual Fantasy Measurement

A further consideration about sexual fantasies regards measurement. Most commonly, sexual fantasies are measured using self-report methodologies, generally with questions centering around the presence and content of fantasies. Common examples of sexual fantasy measures include the Wilson Sex Fantasy Questionnaire [31, 32], the Sexual Fantasy Questionnaire [33], and the Paraphilic Sexual Fantasy Questionnaire [34]. These typically present a series of individual fantasy items (e.g., “Having sex with prepubescent children” or “Having sex with multiple people”), with respondents asked to report how often they experience these fantasies or use them during masturbation.

These measures focus on the content of sexual fantasies, rather than the process of fantasizing as a deliberate act. A range of approaches might thus be used to explore this more deliberative experience of fantasy, in line with the DPM-SF. In support of this notion, Bartels and colleagues reported how having people engage in cognitive tasks (e.g., consciously performing bilateral eye movements) while engaging in sexual fantasizing reduced important markers of the experience of fantasizing [35, 36]. For example, those who engaged in fantasizing while moving their eyes from side-to-side reported how their fantasies were less vivid, less positive, and less arousing than participants who were not asked to engage in eye movements. These data support the DPM-SF distinction between fleeting sexual thoughts (which do not require conscious effort and may be more related simply to fantasy content triggering an attentional shift) on the one hand and deliberative fantasizing on the other.

### Sexual Fantasy and Well-being

With a progressive shift occurring in society towards sexual liberalism [37], sexual fantasies (and sexual fantasizing as a process) are being viewed less frequently as a sign of dissatisfaction with one’s “real” sexual life, but instead as a positive indicator of sexual well-being, freedom, and openness [38–40]. Among other primary human goods, such as excellence in play and happiness, the strengths-based Good Lives Model of offender rehabilitation notes “sexual satisfaction” to be something that all people strive for in life [41, 42]. “Fantasy thinking” (an unrestricted form of mental experiences, in this case of a sexual nature) is thought to encourage arousal, excitement, and a feeling of possibility, feeding into the achievement of sexual satisfaction [43, 44]. Irrespective of relationship status, research suggests that individuals benefit from engagement in an “alternative” sexual reality [45], with this especially being the case for those struggling with sexual dysfunction and physical intimacy [46].

While female “masturbation guilt” has historically been associated with internalized stigma and misogyny [47, 48], the role of masturbation and sexual fantasies as part of healthy sexual development through puberty is being increasingly recognized [49]. Shulman and Horne also noted that masturbation holds the potential to help people to develop improved relationships with their bodies and better recognize their individual sexual needs [50]. Further, masturbation as a positive sexual experience can increase general creativity and moral autonomy [51], with this supporting the view that sexual satisfaction is a primary human good [41]. Research has further demonstrated the benefits of utilizing self-pleasure as a healthy coping strategy for feelings of stress and anxiety [44], with there are also being potential reported benefits for sleep [52]. These data all highlight the positive effects of achieving a sense of sexual fulfillment, and the role that sexual fantasizing can play in the pursuit of this. However, within the context of sexual attractions to children, we must also be mindful of the potential for harm.

### Fantasy and Sexual Offending

A wealth of conflicting evidence exists as to whether there is a relationship between engagement with pornography and sexually aggressive behaviors [53–55]. Although studies have found that men who perceive pornography as realistic are at an increased risk of perpetrating sexual aggression [56], alternative research implies that this relationship is only evident where individuals are predisposed to act aggressively through additional criminogenic factors [57]. It has further been argued that the consumption of non-violent pornographic content correlates with reduced sexual aggression among the general population, with violent pornography having only a weak relationship with sexual aggression [58].

Away from pornography use, there is a small emergent body of literature about the concordance between sexual fantasies and sexual behaviors. The term “concordance” refers to the extent to which one thing (i.e., fantasies) is equivalent to another (i.e., behavior). This emergent work suggests a reasonable amount of concordance between fantasy content and sexual behaviors [59, 60]. However, this relationship tends to be moderated by sex drive, with high sex drive strengthening the fantasy-behavior relationship across a range of legal (e.g., transvestism) and illegal sexual behaviors (e.g., activities involving children or coercion) [59]. One exception to this in Seto and colleagues’ work related to sexual interests in children, where concordance was lower for those with a higher sex drive [59]. This may be related to higher sex drive motivating the seeking of alternative sexual outlets (e.g., with age-appropriate partners), meaning that pedohebephilic behaviors become less pronounced for these individuals within the context of a broader sexual behavioral repertoire. This finding was unable to be replicated in Joyal and Carpentier’s work due to the low number of participants reporting having engaged in sexual activities with children [60].

In more recent work related to the deliberative nature of fantasizing (as per the DPM-SF), Willis and Bartels reported how concordance between fantasizing and fantasy-related behavioral enactment was contingent upon several factors, with the plausibility of enacting the corresponding fantasy behavior being a dominant concern [61]. For those with attractions to children, the illegality of acting on one’s sexual attractions (and associated fantasies) can act as an external/situational inhibitor of engaging in this behavior (thus making it less plausible), with this being further compounded by potential internal inhibitors or a lack of offending facilitators, such as low antisociality [21, 62]. However, people within this group have the same needs for sexual satisfaction as anybody else [41], and as such FSM may provide a viable outlet for their sexual fantasies as they pursue a sense of fulfillment in this domain.

The online availability of child sexual exploitation material (CSEM) is an increasing concern of legislators globally [63], with CSEM being defined as sexually explicit content involving a child or children [64]. As these materials depict the abuse of non-consenting children, the label of “child pornography” is becoming less prevalent in academic discourse due to how this term minimizes the harm caused by creators and users of such material [65, 66]. Merdian and colleagues’ three dimensions model of CSEM offending aims to understand both how and why people engage with CSEM by distinguishing between classifications of users, motivations for offending, and the sociality of their behaviors. According to the model (see also [66, 67]), engagement with CSEM is thought to be driven by sexual fantasies (fantasy-driven) or is used by those with a history of contact sexual offences against children, as a tool to re-live these acts (contact-driven). An individual’s motivations for offending may be driven by a pedophilic sexual interest (with use therefore enabling individuals to achieve sexual gratification) or related to wider deviant sexual interests that are not restricted to children alone [68]. The opportunity to benefit financially through the online exchange of CSEM can also act an offence motivation [69, 70], along with the idea of becoming a “collector” of indecent content with pleasure coming from the organization and categorization of explicit materials [69, 71].

Ward and Keenan argued that an individual’s “implicit theories” can contribute positively or negatively towards their likelihood of engaging with illegal sexual materials [72]. When looking to understand CSEM use specifically, maladaptive schemas (that justify or minimize the act of engaging with such content) are thought to encompass an individual’s beliefs about victims and perceived harm, sexual entitlement, and the world as a hostile environment [73]. CSEM users who are primarily fantasy-driven are thought to display higher levels of sexual deviancy when contrasted against contact-driven users [74]. However, when fantasy was identified as playing a key role in an individual’s engagement with CSEM, Babchishin and colleagues found antisocial behavioral traits to be less prevalent [65, 74].

Although the most common type of CSEM is images and videos of children [75], non-visual materials are also accessible for individuals engaging in fantasy-driven offences [76]. It is these forms of CSEM (which typically do not involve real children) that fall into our definition of FSM. For example, narrative CSEM involves written descriptions of sexual activities with children [77]. The concerns regarding sexual offending using narrative CSEM surround the idea that consuming such content encourages the escalation of fantasies about child abuse, therefore normalizing such behaviors in line with the arguments of social learning theory [78]. Difficulties do however arise when attempting to legally classify alternative forms of CSEM, as the public’s views on perceived harmfulness are thought to be comparatively lower when considering non-visual content, due to the lack of a direct victim [77].

Other forms of FSM, such as human-like sex dolls, have steadily risen in popularity over recent years [79]. However, the evidence about the effects of such objects remains sparse [2, 80]. An initial study aiming to investigate sex doll ownership and aggression found no relationship between ownership and proclivity to engage in sexually aggressive behaviors [81]. These findings are in line with the suggestions of other theorists, in that exposure to pornography may work towards reducing aggressive behaviors via a cathartic effect [82]. Although this initial work focused explicitly on the owners of adult-like sex dolls, subsequent data coming from those who own child-like dolls has revealed a similar pattern of results [83•]. That is, child-like sex doll owners were less likely to express a proclivity for sexual abuse than a comparison group of non-owners who were attracted to children and also demonstrated lower levels of sexual preoccupation. These preliminary data are supportive of a potentially cathartic effect of this form of FSM among those who are attracted to children, which then identifies a potential need for further exploration of similar effects in relation to other forms of this material.

## Applying the Motivation-Facilitation Model to the Use of FSM

The motivation-facilitation model of sexual offending posits that sexual offending occurs when both motivating and facilitating factors are present in an individual and their environment [21]. Suggested motivational factors include paraphilias (e.g., sexual preference disorders such as pedohebephilia), high sex drive (e.g., hypersexuality or sexual compulsivity), and mating effort (i.e., urge to seek out novel sexual stimuli and/or partners). These motivational factors are said to orient an individual towards a propensity for seeking out sexual stimuli. Facilitating factors are those that, when paired together with motivational factors, make it more likely that a sexual offense may occur and can be trait or state in nature. Trait factors may include personality traits (e.g., antisocial or narcissistic personalities), whereas state factors include more environmentally driven features (e.g., drug/alcohol use, access to a potential victim). This model offers a parsimonious explanation of sexual offending that is consistent with the emerging international evidence based on the link between sexual attractions to children and sexual offending. That is, although sexual arousal patterns such as pedophilia may motivate sexual offending among some people, the link is contingent upon the simultaneous presence of facilitating factors. The model can be applied to online offending (e.g., CSEM use) and offline offending (e.g., contact child molestation), with pertinent motivation and facilitation factors being differentially distributed across these two groups [74].

When applying the motivation-facilitation model to the context of FSM use, it can be theorized as to why, for a subset of users, engaging with such material could become problematic and increase the likelihood of committing a child sexual offense (whether that be offline or online), while for others this is not the case. As pedohebephilia is thought to be a motivating factor towards sexual offending in Seto’s model [21], engaging with FSM relating to children could heighten sexual arousal and therefore act as a facilitator to increase offending likelihood. With abstinence from masturbation being self-reported as a risk-management technique by some people who are attracted to children [16], this is a recognized idea by some members of the community. Over time, engaging in CSEM (especially forms such as child-like sex dolls, which offer a more realistic sexual experience) may contribute to the development of offence-supportive beliefs and the adoption of implicit theories about the acceptability of engaging in sexual activity with children (or child-like targets). The combination of enhanced sexual arousal to children (a potential motivator of offending), coupled with the development of permission-giving beliefs (facilitators of offending), may subsequently increase the risk of abuse being committed by somebody with attractions to children.

Alternatively, FSM use could be seen as beneficial by the motivation-facilitation model and instead reduce the likelihood of offending. Rather than heightening arousal, FSM could act as a safe sexual outlet that allows for a feeling of release and sense of catharsis [84], which could reduce a motivation to seek out real children as a sexual partner. Engaging with FSM also avoids the problematic suppression of sexual interests, with such suppression being linked to increases in self-perceived risks for offending among those with attractions to children [15]. In contrast to Stevens and Wood [16], Houtepen and colleagues reported that engagement in masturbatory fantasies was a common coping mechanism used by some people experiencing attractions to children, avoiding the need to access CSEM due to an alternative outlet being identified [3•].

Given the present lack of understanding of FSM and how they are used, it is important to identify the factors associated with use and whether they are risk-enhancing or risk-reducing (i.e., protective). This knowledge could be beneficial to clinicians in the search for more effective methods to support people who are attracted to children when they are seeking help to manage their sexual interests. Nonetheless, Seto’s motivation-facilitation model provides a theoretical framework for thinking about this topic in a more nuanced way [21].

## Setting a Research Agenda for FSM Use by People with Attractions to Children 

An empirical approach will provide researchers, clinicians, policymakers, and the public with sound data upon which to base professional decisions on the use of fantasy and fictional sexual outlets among people who are attracted to children. To set an agenda for researchers to join this burgeoning field, the following broad domains are of immediate relevance to provide a robust foundation for further inquiry:

1. The different perspectives and motivations of FSM users who are attracted to children.
2. The factors theoretically and/or empirically related to risk-enhancing (e.g., sexual fantasy and aggression) and protective (e.g., sexual fantasy and well-being) uses of FSM by those who are attracted to children.
3. How FSM use among those with attractions to children are linked to help-seeking and treatment needs.

### Understanding the Motivations and Experiences of FSM Use

Although existing work on broader sexual fantasies provides some indication as to how fictional materials are used [27], the answers we as researchers develop will always be limited unless we include the perspective of the users. Such research should survey those who are attracted to children to identify what currently is used as a form of FSM, how frequently these materials are used, and what role they that they play in the lives of this population. Comparative work looking at the differences in important outcomes (e.g., well-being, life satisfaction, or risk) in groups who both do and do not engage in FSM use will likely also be a useful first step into this emergent research area.

It is in this endeavor that phenomenological methods are also crucial to providing contextual information about FSM use. This approach takes us beyond surface-level definitions of FSM, or correlational data about frequency of use and levels of hypothetical risk, and towards a more functional account of the use of FSM by those who experience attractions to children. For example, this kind of analysis of how decisions are made to begin engaging with FSM by this population and how FSM use is experienced from a psychosocial and psychosexual perspective (e.g., exploring the interplay between attractions, sexual satisfaction, emotional variables, and interpersonal functioning). Motivational factors can also be probed using such methods. For instance, although many scholars (and legislators) infer deviant sexuality from the ownership of sex dolls [85–88], emergent phenomenological work by Lievesley and colleagues with men who own sex dolls revealed how these individuals often report ownership of dolls as a functional response to perceived deficits in themselves and potential living partners [88]. As such, this work further illuminated a range of motivating factors for doll ownership that are perhaps not obvious when simply observing the materials themselves (for survey data showing such a range of motivations, see [79, 89]). Similar findings may also be found in relation to a broader suite of FSM-related behaviors, with qualitative work being an appropriate method for understanding how users construct and make sense of these actions.

### FSM Use as Risk-Enhancing or Risk-Reducing?

Having established the motivations and perceived functions of FSM among people with attractions to children, it would be logical to subsequently identify those factors that are theoretically and/or empirically related to risk-enhancing and protective uses of FSM. As previously suggested, considering potential links with sexual aggression (as a risk-enhancing factor) or catharsis and improved mental well-being (as protective factors) are the clearest places to start. Many of these potential factors stem from the research highlighted above, particularly those pertaining to CSEM use [78]. It must be considered as to whether these factors also apply to FSM users, addressing whether the pattern is the same or different for a range of forms of FSM.

Given the topic at hand, it will be important to contextualize such work within a theoretical framework that is known to explain sexual offending. Seto’s motivation-facilitation model [21] offers this and provides a parsimonious model within which to explore the links between FSM and sexual offending risk levels. Depending on the research question, FSM can be conceptualized as both a motivating or a facilitating factor. For instance, if one is simply interested in whether FSM use increases (or decreases) risks for engaging in sexual abuse among those with attractions to children, it is possible to build a statistical model that places levels (or, perhaps more accurately, degrees or intensities) of such sexual attractions as a focal predictor of sexual offending risk (i.e., the primary motivator), and FSM use as a moderating variable that adjusts, either positively or negatively, any observed effect (i.e., as a potential facilitating factor). However, if a research team is interested in understanding the effects of FSM on risk in a more direct way, then FSM use itself becomes a focal predictor (i.e., a motivator of offending), with other psychosocial constructs (e.g., antisociality or offense-supportive cognitive distortions) acting as moderator variables (i.e., facilitating factors).

An example experimental design to address this question of exposure to FSM and related behavioral outcomes among healthy controls could involve self-reporting of aggressive behaviors with an additional behavioral test. By controlling for relevant confounds (e.g., age, past offending status, relationship status, living conditions), it can be evaluated whether exposure to (or use of) FSM alters risk indices of risk relative to non-exposure or non-use. Ultimately, however, the aim of this specific program of work should be to understand how, why, and under which conditions FSM use may be risky or safe. This endeavor requires multiple streams of research, using varied methods (e.g., observational studies, cross-sectional and moderation-based analyses, and experimental research) to provide a full picture of the utility of FSM in abuse prevention contexts.

### FSM in Treatment and Help-Seeking Contexts

Although there has been some discussion within clinical spaces about the potential use of sex dolls in therapeutic settings (e.g., in sexual dysfunction, relationship therapy, and preventative settings geared around the reduction of sexual abuse) [60, 90, 91], no empirical work has yet been conducted to explore the efficacy of these (or any other form of FSM) in such contexts. Specifically in relation to people with sexual attractions to children, the relationship between FSM use and help-seeking and treatment needs should be established.

It has recently been reported that some community-based people with sexual attractions to children report a desire to address sexual frustration [26••]. Given that effective treatment engagement involves the alignment of treatment content to service user needs, considering sexual fulfillment and satisfaction as an important treatment need might be an increasingly pressing issue as support services for this population grow in number. However, the effects of using FSM in formal treatment settings are currently unknown.

To assess this within the context of risk, one potential methodology could be to embed FSM use as a tool within existing offending behavior program that target sexual arousal patterns, such as the Healthy Sex Programme in the UK. This allows for a controlled environment to test the effects of FSM with people who may be experiencing offense-related sexual thoughts and feelings, to explore whether usage reduces deviant fantasizing via catharsis-related effects, or if it has a positive effect on indices of potential sexual risk (e.g., sexual preoccupation; [92]). Upon successful evaluation of this as a risk-reducing strategy, community-based options may be made available for further testing.

Treatment alignment (i.e., ensuring congruence in treatment aims between therapists and service users) is also an important consideration, and so exploring the best contexts within which to offer these kinds of services is an additional avenue for future research. Here, qualitative projects with a range of stakeholders (e.g., therapists from general psychological and specialist sex-related services, policymakers, and organizational leaders) may serve an important function in understanding levels of comfort and ability to work with sexual frustration as a treatment need, as well as considering these issues within a defensive decision-making context with people who may be perceived by some professionals as being at an increased risk of causing sexual harms [10, 93].

### Social, Legal, and Ethical Considerations

As alluded to earlier in this paper, the legal landscape related to various forms of FSM is rapidly changing across different jurisdictions. Additionally, those working in professional settings where FSM might be a useful addition to their clinical toolkits fear both public and professional backlash while trying to work ethically. Efforts to think more progressively about the best ways in which to work are often hindered by prominent papers explicitly highlighting a lack of available empirical evidence about the utility of some forms of FSM, but which then call for their avoidance in practice due to potential risks (while ignoring potential benefits) [86, 94].

We are, of course, cognizant of the ethical and legal concerns that people may have about the use of FSM, whether this use is in the community without professional supervision or in formal therapeutic contexts. This is especially difficult when working internationally and remotely in treatment contexts, where there may be a discrepancy between the laws under which a professional and a service user operates. As with other topics in this space, data are needed to better identify where, how, and why different forms of FSM might have a role to play in the treatment and support of people with sexual attractions to children. It is important to survey professionals and policymakers about their views, too, such that these will be the groups using and legislating about the use of FSM. By beginning to work in an exploratory manner, an initial groundswell of data will inform the development of an agenda for both research and policy engagement to better conceptualize how FSM can be used safely, ethically, and legally in the pursuit of both health and fulfillment among those with attractions to children on the one hand, and reduced rates of child sexual abuse on the other.

## Conclusions

Our aim in this conceptual paper has been to explore FSM in relation to emerging ideas on their use among people who experience attractions to children. We considered arguments towards both the risk-enhancing and protective uses of FSM, particularly highlighting the potential for such outlets to benefit people who experience attractions to children who are seeking help, support, or trying to manage their sexual attractions. As our knowledge base expands beyond the prison walls, so does the need to consider a range of treatment options for people with such sexual attractions as they seek happy, healthy, and broadly fulfilling lives. We encourage scholars to follow these questions as we seek a more comprehensive understanding of the best ways to support this group within the community in health and abuse prevention contexts.
